# Pubertal Timing Across Asian American, Native Hawaiian, and Pacific Islander Subgroups

**DOI:** 10.1001/jamanetworkopen.2024.10253

**Published:** 2024-05-13

**Authors:** Ai Kubo, Julia Acker, Sara Aghaee, Lawrence H. Kushi, Charles P. Quesenberry, Louise C. Greenspan, Shylaja Srinivasan, Alka M. Kanaya, Julianna Deardorff

**Affiliations:** 1 Kaiser Permanente Division of Research, Oakland, California; 2 School of Public Health, University of California, Berkeley; 3 Kaiser Permanente San Francisco Medical Center, San Francisco, California; 4 Division of Pediatric Endocrinology, Department of Pediatrics, University of California, San Francisco; 5 Division of General Internal Medicine, Department of Medicine, University of California, San Francisco

## Abstract

**Importance:**

Earlier puberty is associated with adverse health outcomes, such as mental health issues in adolescence and cardiometabolic diseases in adulthood. Despite rapid growth of the Asian American, Native Hawaiian, and Pacific Islander populations in the US, limited research exists on their pubertal timing, potentially masking health disparities.

**Objective:**

To examine pubertal timing among Asian American, Native Hawaiian, and Pacific Islander children and adolescents by disaggregating ethnic subgroups.

**Design, Setting, and Participants:**

This retrospective cohort study included Asian American, Native Hawaiian, and Pacific Islander youths aged 5 to 18 years assessed for pubertal development at Kaiser Permanente Northern California, a large, integrated health care delivery system. Follow-up occurred from March 2005, through December 31, 2019. Data were analyzed in October 2023.

**Exposure:**

Race and ethnicity, categorized into 11 ethnic subgroups: Asian Indian, Chinese, Filipino, Japanese, Korean, Native Hawaiian and Pacific Islander, Other South Asian, Other Southeast Asian, Vietnamese, multiethnic, and multiracial.

**Main Outcomes and Measures:**

Pubertal timing was determined using physician-assessed sexual maturity ratings (SMRs). Outcomes included the median age at transition from SMR 1 (prepubertal) to SMR 2 or higher (pubertal) for onset of genital development (gonadarche) in boys, breast development (thelarche) in girls, and pubic hair development (pubarche) in both boys and girls.

**Results:**

In this cohort of 107 325 Asian American, Native Hawaiian, and Pacific Islander children and adolescents (54.61% boys; 12.96% Asian Indian, 22.24% Chinese, 26.46% Filipino, 1.80% Japanese, 1.66% Korean, 1.96% Native Hawaiian and Pacific Islander, 0.86% Other South Asian, 3.26% Other Southeast Asian, 5.99% Vietnamese, 0.74% multiethnic, and 22.05% multiracial), the overall median ages for girls’ pubarche and thelarche were 10.98 years (95% CI, 10.96-11.01 years) and 10.13 years (95% CI, 10.11-10.15 years), respectively. For boys’ pubarche and gonadarche, median ages were 12.08 years (95% CI, 12.06-12.10 years) and 11.54 years (95% CI, 11.52-11.56 years), respectively. Differences between subgroups with earliest and latest median age at onset were 14 months for girls’ pubarche, 8 months for thelarche, 8 months for boys’ pubarche, and 4 months for gonadarche. In general, Asian Indian, Native Hawaiian and Pacific Islander, and Other South Asian subgroups had the earliest ages at onset across pubertal markers, while East Asian youths exhibited the latest onset. Restricting to those with healthy body mass index did not substantially change the findings.

**Conclusions and Relevance:**

In this cohort study of Asian American, Native Hawaiian, and Pacific Islander children and adolescents, pubertal timing varied considerably across ethnic subgroups. Further investigation is warranted to assess whether these differences contribute to observed health disparities in adulthood, such as type 2 diabetes and cardiovascular diseases.

## Key Points

**Question:**

Does pubertal timing vary among Asian American, Native Hawaiian, and Pacific Islander youths when disaggregated by ethnic subgroups?

**Findings:**

In this cohort study of 107 325 US children and adolescents, substantial variations in pubertal timing across Asian American, Native Hawaiian, and Pacific Islander ethnic subgroups were identified. Asian Indian, Native Hawaiian and Pacific Islander, and Other South Asian youths typically had earlier ages at pubertal onset, while Chinese and Korean youths exhibited later onset.

**Meaning:**

These findings may provide insight into disparities in chronic diseases, such as type 2 diabetes and cardiovascular diseases, later in life.

## Teaser

This cohort study investigates whether there are differences in pubertal timing among Asian American, Native Hawaiian, and Pacific Islander individuals after disaggregating by ethnic subgroups.

## Introduction

Globally, girls are experiencing earlier pubertal onset today than in past generations.^1^ This is a significant public health concern because early puberty in girls has been linked to a variety of behavioral and emotional problems in adolescence^2,3,4,5,6^ and serious chronic conditions later in life, including cancers,^7,8^ type 2 diabetes (T2D),^9^ and cardiovascular diseases.^10^ Fewer studies have been conducted among boys, although they too seem to be experiencing earlier pubertal timing,^11,12^ with associated increased risks of externalizing behaviors during adolescence^2,4,6^ and of cardiovascular diseases, diabetes, and prostate and testicular cancers later in life.^3,10,13,14,15,16^

In the US, there are marked differences in pubertal timing across racial and ethnic groups, with studies consistently showing that non-Hispanic Black (hereafter, *Black*) and Hispanic or Latino youths experience puberty significantly earlier than non-Hispanic White (hereafter, *White*) youths.^11,17^ Very little is known, however, regarding the pubertal timing among Asian American, Native Hawaiian, and Pacific Islander youths even though these populations are among the fastest-growing groups in the US.^18^ Previous US studies either did not include these groups or, if they did, aggregated Asian American subgroups and Native Hawaiian and Pacific Islander youths into 1 group. Aggregation of ethnic groups that have distinct health-related characteristics may mask disparities in health outcomes.^19^ Given the associations between early puberty and various health outcomes later in life, examining variations in the timing of puberty across Asian American, Native Hawaiian, and Pacific Islander subgroups could provide insights into early health indicators underlying disparities in chronic conditions.

To address these significant gaps in the literature, we assessed pubertal timing among Asian American, Native Hawaiian, and Pacific Islander youths by disaggregating ethnic subgroups using a large, diverse sample of boys and girls receiving care in an integrated health care delivery system. As obesity is one of the most robust factors associated with early pubertal timing, especially among girls,^17,20,21^ we conducted analyses for the overall sample and, as a sensitivity analysis, only among individuals with healthy weight.

## Methods

### Setting

Kaiser Permanente Northern California (KPNC) is an integrated health care delivery system with over 4.4 million members and comprises approximately 29% of the child and adolescent (aged 10-19 years) northern California population.^22^ The KPNC membership is racially and ethnically diverse and is similar sociodemographically to the overall population.^22^ The KPNC institutional review board approved this study with a waiver of the requirement for informed consent because the study was based on data extracted from electronic health records (EHRs) with no participant contact. This cohort study followed the Strengthening the Reporting of Observational Studies in Epidemiology (STROBE) reporting guideline.

### Participants

The EHR was used to identify Asian American, Native Hawaiian, and Pacific Islander youths who had been assessed for pubertal development during routine pediatric primary care appointments at a KPNC facility. Data were also extracted from other KPNC clinical and administrative databases, including the KPNC Division of Research Virtual Data Warehouse, which harmonizes data across various data sources.^23^ Follow-up for determining pubertal development began in March 2005 and continued through December 31, 2019. Data after 2019 were excluded due to disruptions in primary care from the COVID-19 pandemic as well as emerging evidence suggesting the pandemic may be associated with earlier onset of puberty in some populations.^24^ Individuals with a pubertal assessment documented in the EHR before age 5 years or after age 18 years were excluded from the study. Additionally, youths with a nonspecific race and ethnicity designation (eg, *Asian*) were excluded.

### Measurements

#### Race and Ethnicity

Race and ethnicity are typically documented in the EHR based on self-reported information from the KPNC member and may come from multiple source databases, including demographic data collected at clinic visits and during health plan enrollment. We categorized individuals into 9 ethnic subgroups: Asian Indian, Chinese, Filipino, Japanese, Korean, Native Hawaiian and Pacific Islander, Other South Asian, Other Southeast Asian, and Vietnamese. Additionally, youths identifying with more than 1 of these subgroups were categorized as multiethnic. Those identifying as Asian American, Native Hawaiian, and/or Pacific Islander in combination with another race (ie, American Indian or Alaska Native, Black, Hispanic or Latino, or White) were classified as multiracial. Because the focus of the analyses was to describe pubertal timing across Asian American, Native Hawaiian, and Pacific Islander individuals, we did not include other major racial and ethnic groups (eg, White, Black, or Hispanic or Latino). These groups also exhibit marked heterogeneity, and the undertaking to disaggregate each of these was beyond the scope of the current study.

#### Pubertal Timing

Physician-assessed sexual maturity ratings (SMRs or Tanner stages)^25^ are a routine part of KPNC pediatric appointments for children aged 6 years or older. SMRs use a 5-point ranking system to measure pubertal development from prepuberty (SMR 1) to full maturation (SMR 5). For girls, breast stage was assessed using visual inspection and palpation of breast tissue (to avoid miscategorization due to adiposity). For boys, testicular and genital stage was assessed using visual inspection and palpation of testicular tissue to estimate testicular volume. Pubic hair stage was assessed using visual inspection for both sexes. The accuracy of KPNC SMRs was validated and described in a previous study.^21^ In this study, outcomes of interest were age at transition from SMR 1 (prepubertal) to SMR 2 or higher (pubertal) for genital development onset (gonadarche) in boys, breast development onset (thelarche) in girls, and pubic hair development onset (pubarche) in both boys and girls.

#### Childhood Body Mass Index

Child weight and height measurements were obtained from clinic visits between the ages of 5 and 6 years to estimate body mass index (BMI) prior to pubertal onset. We chose the weight and height closest to the 5th birthday to calculate BMI percentiles using age- and sex-specific Centers for Disease Control and Prevention year 2000 standard population distributions.^26^ Body mass index was classified into underweight (less than 5th percentile), normal weight (5th to <85th percentile), overweight (85th to <95th percentile), and obese (≥95th percentile) categories.

### Statistical Analysis

Interval-censored survival analysis was used to estimate the timing of pubertal onset, which accounts for the transitions that occur in SMR stages between pediatric checkups. Youths were considered interval-censored if examined at SMR 1 and then at SMR 2 or higher at a subsequent visit. Youths were left-censored if they had already transitioned to SMR 2 or higher at the time of their first examination with an SMR assessment and right-censored if they had not transitioned to SMR 2 or higher by the end of follow-up.

Maximum likelihood estimates of the median age at pubertal onset were estimated overall and separately for each ethnic subgroup by sex, assuming a Weibull distribution for time to event. Monte Carlo 95% CIs for the median ages were estimated based on 4000 random samples from the asymptotic normal distribution of the maximum likelihood estimators of the Weibull distribution shape and scale parameters. We also conducted sensitivity analyses restricting to only those with BMIs in the 5th to less than 85th percentiles to describe the differences in pubertal timing across ethnic subgroups independent of the prevalence of childhood overweight and obesity. Analyses were conducted in October 2023 using the icenReg package, version 2.0.15, in R, version 4.2.2 (R Project for Statistical Computing).^27^

## Results

### Participants

The analytic cohort included 107 325 youths, consisting of 58 613 boys (54.61%) and 48 712 girls (45.39%). A total of 12.96% were Asian Indian; 22.24%, Chinese; 26.46%, Filipino; 1.80%, Japanese; 1.66%, Korean; 1.96%, Native Hawaiian and Pacific Islander; 0.86%, Other South Asian; 3.26%, Other Southeast Asian; 5.99%, Vietnamese; 0.74%, multiethnic; and 22.05%, multiracial. We initially identified 113 399 KPNC patients aged 5 to 18 years who had an SMR and had a disaggregated Asian American or Native Hawaiian and Pacific Islander subgroup specified. Among them, 5559 were excluded due to medical conditions that may affect pubertal development (eg, congenital adrenal hyperplasia). Additionally, 515 were excluded due to regression in SMR stages over time, meaning that individuals went from pubertal at one visit to prepubertal at a later visit. The distribution of ethnic subgroups in the final sample is described in the Table.

**Table.** Distribution of Ethnic Subgroups Among Asian American, Native Hawaiian, and Pacific Islander Boys and Girls Assessed for Pubertal Development at Kaiser Permanente Northern California, 2005-2019

Ethnic subgroup	Participants, No. (%)		
	Total (N = 107 325)	Boys (n = 58 613)	Girls (n = 48 712)
Asian Indian	13 911 (12.96)	7980 (13.61)	5931 (12.18)
Chinese	23 874 (22.24)	12 677 (21.63)	11 197 (22.99)
Filipino	28 399 (26.46)	15 464 (26.38)	12 935 (26.55)
Japanese	1937 (1.80)	1036 (1.77)	901 (1.85)
Korean	1785 (1.66)	1016 (1.73)	769 (1.58)
Native Hawaiian and Pacific Islander	2107 (1.96)	1201 (2.05)	906 (1.86)
Other South Asian	920 (0.86)	518 (0.88)	402 (0.83)
Other Southeast Asian	3501 (3.26)	1925 (3.28)	1576 (3.24)
Vietnamese	6432 (5.99)	3529 (6.02)	2903 (5.96)
Multiethnic^a^	796 (0.74)	461 (0.79)	335 (0.69)
Multiracial^b^	23 663 (22.05)	12 806 (21.85)	10 857 (22.29)

^a^ Refers to identification with multiple Asian American, Native Hawaiian, and/or Pacific Islander ethnic subgroups.

^b^ Refers to identification with Asian American, Native Hawaiian, and/or Pacific Islander subgroups in combination with another racial or ethnic group (ie, American Indian or Alaska Native, Black, Hispanic or Latino, or White).

### Pubarche in Girls

**Figure 1.**
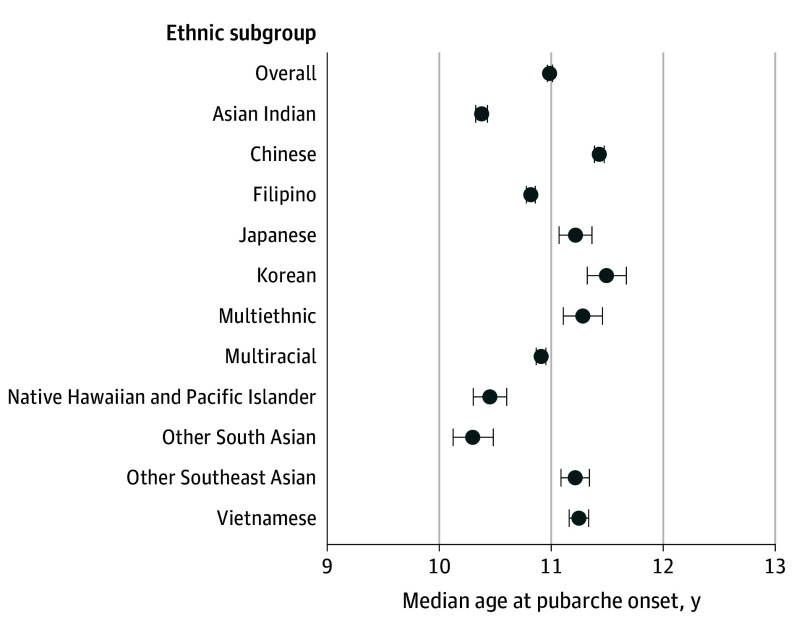
Median Age at Pubarche in Girls by Asian American, Native Hawaiian, and Pacific Islander Ethnic Subgroups Multiethnic refers to identification with multiple Asian American, Native Hawaiian, and/or Pacific Islander ethnic subgroups. Multiracial refers to identification with Asian American, Native Hawaiian, and/or Pacific Islander subgroups in combination with another racial or ethnic group (ie, American Indian or Alaska Native, Black, Hispanic or Latino, or White). Horizontal lines represent 95% CIs.

The pubarche analysis included 47 500 girls. The overall median age at pubarche among aggregated Asian American, Native Hawaiian, and Pacific Islander groups was 10.98 years (95% CI, 10.96-11.01 years). When we disaggregated the study population into ethnic subgroups, we observed stark variations in the timing of pubarche across ethnic subgroups (Figure 1). Notably, Other South Asian (10.30 years; 95% CI, 10.12-10.48 years), Asian Indian (10.38 years; 95% CI, 10.33-10.43 years), and Native Hawaiian and Pacific Islander (10.45 years; 95% CI, 10.30-10.60 years) girls exhibited the earliest median ages at pubarche. In contrast, Korean girls had a substantially later median pubarche at 11.49 years (95% CI, 11.32-11.67 years); the difference in the median age at pubarche between Other South Asian and Korean girls was 14 months.

### Thelarche in Girls

**Figure 2.**
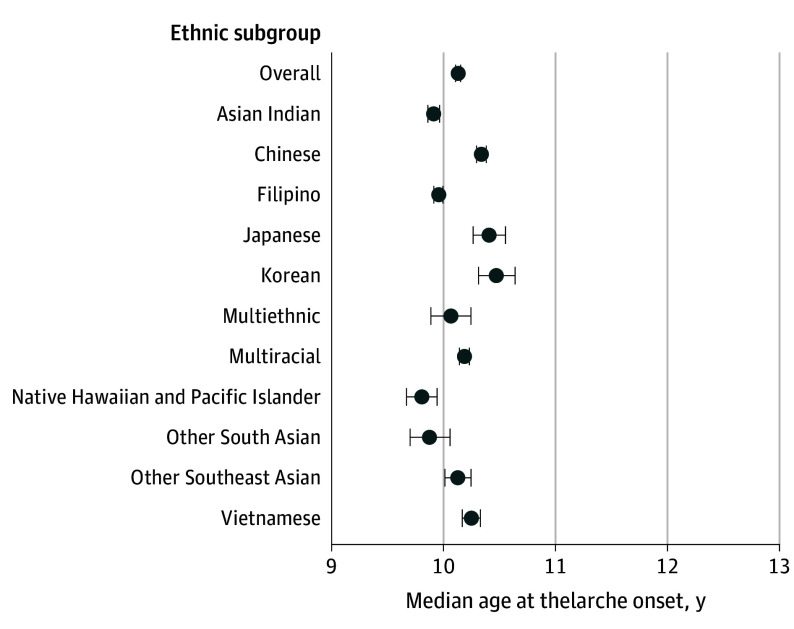
Median Age at Thelarche in Girls by Asian American, Native Hawaiian, and Pacific Islander Ethnic Subgroups Multiethnic refers to identification with multiple Asian American, Native Hawaiian, and/or Pacific Islander ethnic subgroups. Multiracial refers to identification with Asian American, Native Hawaiian, and/or Pacific Islander subgroups in combination with another racial or ethnic group (ie, American Indian or Alaska Native, Black, Hispanic or Latino, or White). Horizontal lines represent 95% CIs.

The overall median age at thelarche (n = 47 592) was 10.13 years (95% CI, 10.11-10.15 years), with significant variations in the timing across subgroups (Figure 2). Native Hawaiian and Pacific Islander girls had the earliest median age at thelarche at 9.80 years (95% CI, 9.67-9.94 years) followed by Other South Asian girls at 9.87 years (95% CI, 9.70-10.06 years). In contrast, Korean girls had the latest median onset at 10.47 years (95% CI, 10.31-10.64 years), which was 8 months later than their Native Hawaiian and Pacific Islander counterparts.

### Pubarche in Boys

**Figure 3.**
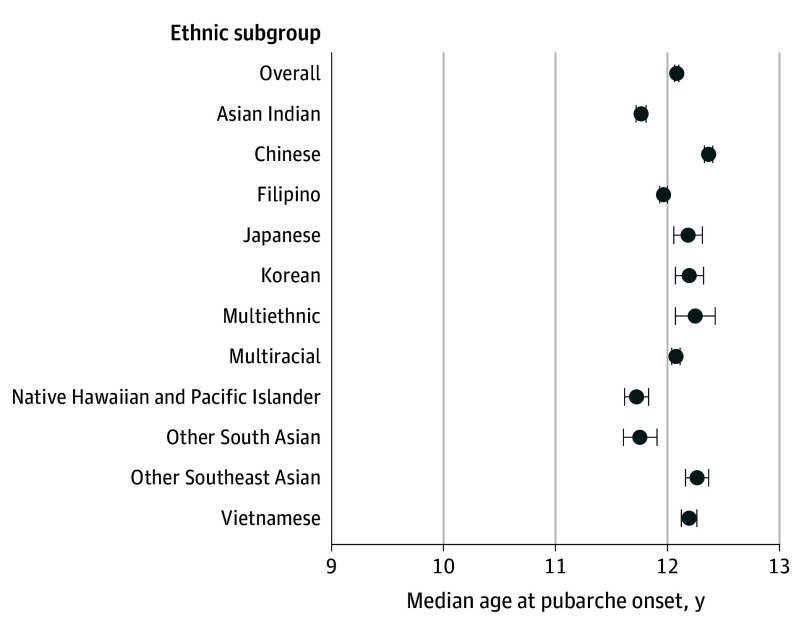
Median Age at Pubarche in Boys by Asian American, Native Hawaiian, and Pacific Islander Ethnic Subgroups Multiethnic refers to identification with multiple Asian American, Native Hawaiian, and/or Pacific Islander ethnic subgroups. Multiracial refers to identification with Asian American, Native Hawaiian, and/or Pacific Islander subgroups in combination with another racial or ethnic group (ie, American Indian or Alaska Native, Black, Hispanic or Latino, or White). Horizontal lines represent 95% CIs.

The overall median age at pubarche for boys among aggregated Asian American, Native Hawaiian, and Pacific Islander groups (n = 58 295) was 12.08 years (95% CI, 12.06-12.10 years). Subgroup variations were observed when data were disaggregated (Figure 3); pubarche in boys was earliest among Native Hawaiian and Pacific Islander (11.72 years; 95% CI, 11.62-11.83 years), Other South Asian (11.75 years; 95% CI, 11.61-11.91 years), and Asian Indian (11.77 years; 95% CI, 11.72-11.81 years) boys. In contrast, Chinese boys had the latest median pubarche at 12.37 years (95% CI, 12.33-12.40 years). The difference in median age at pubarche between Native Hawaiian and Pacific Islander boys and Chinese boys was 8 months.

### Gonadarche in Boys

**Figure 4.**
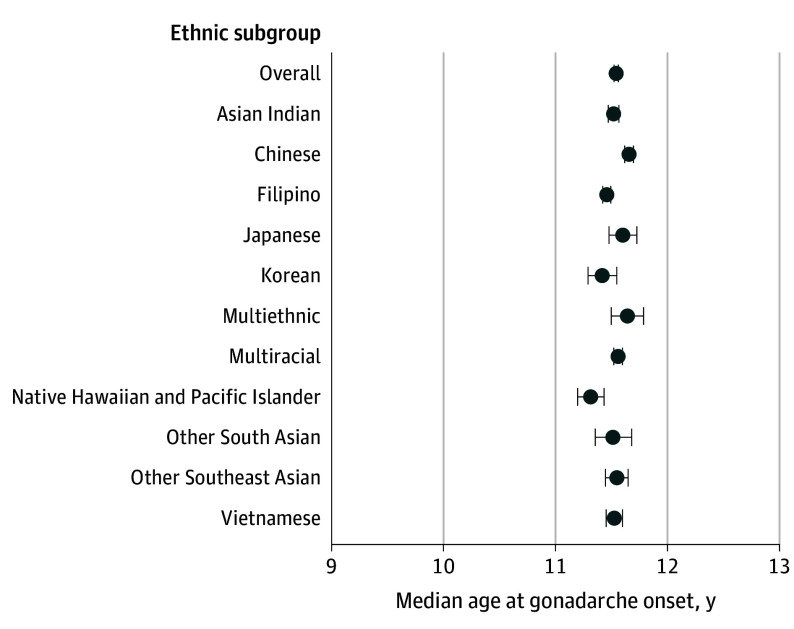
Median Age at Gonadarche in Boys by Asian American, Native Hawaiian, and Pacific Islander Ethnic Subgroups Multiethnic refers to identification with multiple Asian American, Native Hawaiian, and/or Pacific Islander ethnic subgroups. Multiracial refers to identification with Asian American, Native Hawaiian, and/or Pacific Islander subgroups in combination with another racial or ethnic group (ie, American Indian or Alaska Native, Black, Hispanic or Latino, or White). Horizontal lines represent 95% CIs.

The overall median age at gonadarche in boys (n = 57 373) was 11.54 years (95% CI, 11.52-11.56 years). In contrast to the other pubertal markers, gonadarche in boys displayed substantially less variability across the ethnic subgroups (Figure 4). The difference between the earliest- and latest-onset groups was 4 months (Native Hawaiian and Pacific Islander boys at 11.31 years [95% CI, 11.20-11.43 years] compared with Chinese boys at 11.66 years [95% CI, 11.62-11.70 years]).

### Sensitivity Analyses

When we restricted the cohort to those who were in the healthy weight category (5th to <85th BMI percentile) at age 5 to 6 years, the significant variations in the timing of pubertal onset across subgroups persisted, although the 95% CIs widened slightly due to the smaller sample sizes. Asian Indian, Native Hawaiian and Pacific Islander, and Other South Asian girls continued to have the earliest onset of pubarche and thelarche, while Chinese, Japanese, Korean, and Vietnamese girls had substantially later onset. For instance, the difference between the median ages of pubarche between Asian Indian girls and Chinese girls was 14 months. Among boys, Native Hawaiian and Pacific Islander boys continued to have the earliest pubarche and gonadarche among all the subgroups, while Chinese boys had the latest onset.

## Discussion

To our knowledge, this is the first population-based study to describe pubertal timing among Asian American, Native Hawaiian, and Pacific Islander boys and girls in the US and the only one to compare differences in timing by disaggregating the population by several Asian American subgroups. We found substantial differences in the timing of pubertal onset across ethnic subgroups; Asian Indian, Native Hawaiian and Pacific Islander, and Other South Asian boys and girls tended to experience earlier pubertal onset than their East Asian counterparts. The variability of pubertal timing across ethnic subgroups was greater for girls than for boys. These differences persisted even when we restricted the samples to those with a healthy BMI. These findings suggest that other factors in addition to BMI likely contribute to disparities in pubertal timing, such as stress, environment, and lifestyle factors (eg, diet, physical activity).

About 7.2% of US individuals, or 24 million people, identify as being of Asian descent, and another 0.5% are of Native Hawaiian and Pacific Islander origin.^28^ Asian American, Native Hawaiian, and Pacific Islander groups are among the most rapidly growing populations in the US^18^; the Asian American population is projected to surpass 46 million by 2060.^18^ Although many studies have described secular trends toward earlier timing of puberty among US children and adolescents, few studies included Asian American, Native Hawaiian, and Pacific Islander populations. For instance, the landmark 1997 study by Herman-Giddens et al,^29^ often used as baseline data when describing contemporary trends in pubertal timing in the US, included only Black and White girls. A follow-up study from this group described pubertal timing among boys^11^ and included Black, Hispanic, and White boys but not Asian American or Native Hawaiian and Pacific Islander boys. One of the few studies of pubertal timing that included Asian American children was conducted by Biro and colleagues,^17^ who concluded that Asian American and White girls had later onset of breast development compared with their Black or Hispanic counterparts. However, only 57 girls from this study (5%) were Asian American, with few Native Hawaiian and Pacific Islander girls; no disaggregated data for Asian American subgroups or specific rates for the Native Hawaiian and Pacific Islander population were reported.

Additional research specifically addressing pubertal timing among Asian American, Native Hawaiian, and Pacific Islander children and adolescents is limited to menarche data with no examination by ethnic subgroup. For instance, findings from the National Longitudinal Study of Adolescent Health suggested that Asian American girls are less likely to experience early menarche (≤11 years) and more likely to have late menarche (≥14 years) compared with their peers of other ethnicities.^30^ The authors noted that Chinese and Filipino girls constituted a significant portion of the Asian American sample but did not specify whether Native Hawaiian and Pacific Islander girls were included. Conversely, a cohort study of 1386 girls from Catholic schools in Los Angeles reported that Asian American, Native Hawaiian, and Pacific Islander girls (n = 164) reached menarche at a median age of 12.2 years compared with 12.8 years for White girls.^31^ However, the generalizability of this study is questionable due to its specific population.

Our study extends the existing literature by demonstrating marked variability across Asian American, Native Hawaiian, and Pacific Islander subgroups. It is known that aggregation of heterogeneous subgroups that have distinct health-related cultural and lifestyle differences likely results in masking of risk estimation.^19^ This study highlights the importance of disaggregating data to potentially unmask important social and health differences to better inform health policies and resource allocation that are tailored to the needs of specific subgroups.^32,33^ Further investigation to better understand the sources of health disparities across Asian American, Native Hawaiian, and Pacific Islander subgroups is critical and may elucidate targets that are subgroup specific for preventive interventions to address certain disease outcomes.

Health disparities in chronic conditions are well documented among Asian American, Native Hawaiian, and Pacific Islander subgroups. Some disparities in pubertal development may correspond to disparities in chronic diseases later in life, such as T2D, cardiovascular diseases,^34^ and cancer.^35^ For example, a study on adult T2D revealed that Filipino, Native Hawaiian and Pacific Islander, and South Asian individuals had the highest prevalence and incidence among all racial and ethnic groups.^36^ Similar results have been reported on gestational diabetes.^37^ Our results are in line with these disparities. Early pubarche has been found to be associated with increased risks of cardiometabolic diseases, including T2D and gestational diabetes.^9,38^ Further research is needed to determine whether the disparities in pubertal timing observed in this study correspond to the disparities in T2D and gestational diabetes among these subgroups in adult populations.

### Strengths and Limitations

Marked strengths of our study are the inclusion of boys and the use of objective measures of puberty assessed by pediatricians. Most previous studies of pubertal timing only included girls and relied solely on menarche data. Using menarche data as the only marker of puberty poses methodologic problems. Pubertal transitions take place over several years: among girls, for instance, pubertal characteristics often start with thelarche followed by pubarche, acne, a growth spurt, and then menarche. Other biological maturation events subsequently occur (eg, full or adult breasts or pubic hair growth). In addition, boys have remained vastly understudied because there is no similar hallmark of puberty that can be measured reliably. The observable signs of puberty in boys often begin with gonadarche (testicular enlargement and penile growth) followed by spermarche, pubarche, and later, the growth spurt, acne, voice deepening, and facial hair.^39^ Previous studies have used recalled age at shaving initiation,^40^ age at first nocturnal emission,^41,42^ and age at voice change or cracking^16,41,42,43^ to assess onset. These are far from the best proxies for age at initiation or completion of the pubertal process and are subject to recall bias. Because of the availability of pediatrician-assessed SMRs, we were able to include boys, and results demonstrated that Asian American, Native Hawaiian, and Pacific Islander boys had less variability in pubertal timing across ethnic subgroups compared with girls.

There are also some study limitations. First, because our data were collected through routine pediatric visits and not for research purposes, we did not have information on diet, exercise, other lifestyle factors, and family history that may have contributed to differences in pubertal timing. However, availability of EHR data and a large and diverse cohort of Asian American, Native Hawaiian, and Pacific Islander boys and girls provided us with a unique opportunity to disaggregate the data by ethnic subgroups, enabling the study to be the first to focus on pubertal timing among these populations, to our knowledge. Second, we used SMRs assessed primarily by pediatricians rather than more highly trained endocrinologists; thus, staging may be less accurate. However, our group conducted a validation study (described elsewhere^21^), which demonstrated high correlation between pediatrician- and pediatric endocrinologist–assessed SMRs, suggesting that the data are relatively reliable. Many previous studies assessed pubertal timing by self- or parental report; objective measures of pubertal assessment by clinicians is thus an important strength of this study. Third, we were unable to further disaggregate participants in the multiethnic or multiracial groups. There are many combinations of race and ethnicity comprising these groups, making it difficult to determine how to disaggregate the groups in a way that would permit us to draw meaningful conclusions from the analyses. However, we included these groups in our analyses to better represent the population of northern California.

## Conclusions

In this cohort study of 107 325 Asian American, Native Hawaiian, and Pacific Islander children and adolescents from northern California, the median age at pubertal onset varied substantially across ethnic subgroups, even among youths with healthy weight. Further investigation is warranted to assess whether these subgroup differences in pubertal timing correspond to disparities observed in adult chronic conditions, such as T2D.

## Notes

Supplement.
Data Sharing Statement
