# Supplementary material for: Pubertal Timing Across Asian American, Native Hawaiian, and Pacific Islander Subgroups
Source: JAMA Netw Open. 2024 May 13;7(5):e2410253. doi: 10.1001/jamanetworkopen.2024.10253 (PMC11091761; doi:10.1001/jamanetworkopen.2024.10253)
Supplement: Supplement. — Data Sharing Statement [file jamanetwopen-e2410253-s001.pdf]

Data Sharing Statement

Kubo. Pubertal Timing Across Asian American, Native Hawaiian, and Pacific Islander Subgroups. *JAMA Netw Open*. Published May 13, 2024.
doi:10.1001/jamanetworkopen.2024.10253

Data
Data available: No

Additional Information
Explanation for why data not available: The datasets generated and/or analyzed during the current study are not publicly available due to our institutional policy. Individuals who are interested in accessing the data may contact the corresponding author regarding [or to discuss or set up] a data use agreement.
